# LDSplitDB: a database for studies of meiotic recombination hotspots in MHC using human genomic data

**DOI:** 10.1186/s12920-018-0351-0

**Published:** 2018-04-20

**Authors:** Jing Guo, Hao Chen, Peng Yang, Yew Ti Lee, Min Wu, Teresa M. Przytycka, Chee Keong Kwoh, Jie Zheng

**Affiliations:** 1 School of Computer Science and Engineering, Nanyang Technological University, Nanyang Ave, Singapore, 639798 Singapore; 2 Institute for Infocomm Research, Agency for Science, Technology & Research, 1 Fusionopolis Way, Singapore, 138632 Singapore; 3 Genome Institute of Singapore, Agency for Science, Technology, and Research, Biopolis, Singapore, 138672 Singapore; 4 NCBI, NLM, National Institutes of Health, 8600 Rockville Pike, Bethesda, Maryland, 20894 USA

**Keywords:** 1000 Genomes Project, MHC, Meiotic recombination hotspot, DNA sequence polymorphism, GWAS, Epigenetic modification

## Abstract

**Background:**

Meiotic recombination happens during the process of meiosis when chromosomes inherited from two parents exchange genetic materials to generate chromosomes in the gamete cells. The recombination events tend to occur in narrow genomic regions called recombination hotspots. Its dysregulation could lead to serious human diseases such as birth defects. Although the regulatory mechanism of recombination events is still unclear, DNA sequence polymorphisms have been found to play crucial roles in the regulation of recombination hotspots.

**Method:**

To facilitate the studies of the underlying mechanism, we developed a database named LDSplitDB which provides an integrative and interactive data mining and visualization platform for the genome-wide association studies of recombination hotspots. It contains the pre-computed association maps of the major histocompatibility complex (MHC) region in the 1000 Genomes Project and the HapMap Phase III datasets, and a genome-scale study of the European population from the HapMap Phase II dataset. Besides the recombination profiles, related data of genes, SNPs and different types of epigenetic modifications, which could be associated with meiotic recombination, are provided for comprehensive analysis. To meet the computational requirement of the rapidly increasing population genomics data, we prepared a lookup table of 400 haplotypes for recombination rate estimation using the well-known LDhat algorithm which includes all possible two-locus haplotype configurations.

**Conclusion:**

To the best of our knowledge, LDSplitDB is the first large-scale database for the association analysis of human recombination hotspots with DNA sequence polymorphisms. It provides valuable resources for the discovery of the mechanism of meiotic recombination hotspots. The information about MHC in this database could help understand the roles of recombination in human immune system.

**Database URL:**

http://histone.scse.ntu.edu.sg/LDSplitDB

## Background

The major histocompatibility complex (MHC) spans over 3 Mb on the short arm of chromosome 6 in the human genome. It is one of the most important regions in the human genome with respect to immunity. There are more than 200 genes in the MHC region most of which participate in immune responses, and these genes contain many polymorphic loci [1]. The high polymorphism in the MHC segment ensures a diversity of the MHC molecules among individuals and populations [2, 3]. Most of the loci are associated with inflammatory and autoimmune diseases [2, 4], e.g. a variation in MHC class I gene *HFE* causes haemochromatosis [5]. On the other hand, a constant shuffling of haplotypes is potentially beneficial in response to the immune evasion by pathogens, thereby protecting humans from the pathogens [4, 6].

Meiotic recombination is the process of chromosomal crossover leading to the generation of new haplotypes in the gamete cells. The locations where such exchanges occur tend to cluster in short segments of chromosome known as “recombination hotspots”. Efforts have been made over the past two decades to generate informative linkage disequilibrium (LD) maps for the studies of genetic diversity and disease association in MHC [6–10]. Sperm typing technology can be used to classify and estimate the proportion of individual sperms as recombinant or non-recombinant for a short genomic region [11]. Thus it can be used to estimate recombination frequency for a few hotspots in a human individual. For example, detection of meiotic recombination sites in the MHC has been conducted using sperm typing which generated a reliable estimate of the frequency and distribution of recombination events across a segment of DNA [6]. Six hotspots were found to account for 94% of the recombination events in the MHC [7]. These hotspots offer insights into the LD block structure, which is crucial for identifying genes involved in inheritable human diseases [8]. However, despite the high resolution and accuracy, sperm typing tends to be restricted to short genomic regions, and there is still a lack of detailed information about recombination across the entire MHC region.

Single nucleotide polymorphism (SNP) is one of the most easily obtained and common types of genetic variation. The development of genomic sequencing technology provides the opportunity to genotype or sequence a large number of human genomes, e.g. the 1000 Genomes Project and the International HapMap Project which provide valuable information resources. Taking advantage of the sequencing technology, researchers have used the vast amount of admixed population-genotyped SNP data to study the recombination hotspots [9, 10, 12]. For the MHC region, there is marked divergence in the haplotypic structure across different populations [13]. Zheng et al. [14] suggested that there could exist meaningful associations between hotspot strengths and DNA sequence polymorphisms using the LDsplit algorithm [15]. It was observed that the recombination rates in the same hotspot could be significantly different between SNP alleles, e.g. at the *DNA2* hotspot, an approximately 20 folds difference was observed between different samples of chromosomes [14]. Individual-specific recombination rates can be measured or inferred as a molecular phenotype to be associated with genotypes thereby shedding lights on the regulatory mechanisms of meiotic recombination hotspots [12, 16].

In this paper, we present a database named “LDSplitDB”, which is the first database designed for the large-scale association studies of meiotic recombination hotspots. In comparison with other similar databases, e.g. HUMHOT [17], ReDB [18], LDSplitDB presents several distinctive features. First, it is the first database that provides variations in recombination profiles between sub-populations. Secondly, it was designed for the hotspot-SNP association studies for the discovery of recombination mechanisms. Thirdly, auxiliary information, e.g. epigenetic data, has been integrated with cross-link information. To construct LDSplitDB, we estimated the allele-specific recombination strengths in the MHC region using datasets from the HapMap Phase III data and the 1000 Genomes Project, and performed a genome-wide association study (GWAS) of recombination events in all autosomes of European populations from the HapMap Phase II data. Therefore, LDSplitDB offers comprehensive data resources for revealing the mechanisms of the meiotic recombination, discovering haplotypic structures in the MHC region and studying the associations between genetic variation and diseases. The information about different types of epigenetic modifications (from ENCODE [19] and the NIH Roadmap Epigenomics Data Collection [20]), gene locations and the average recombination profiles are also integrated for cross-sectional analysis. The existing lookup tables with at most 192 haplotypes provided by the authors of LDhat [21] cannot meet the needs of computing large datasets of genetic variations, such as those from either the HapMap Phase III or the 1000 Genomes Project. Thus, in LDSplitDB we provide a lookup table for up to 400 haplotypes including all possible two-locus haplotype configurations using high-performance computing (HPC).

## Construction and content

### Data preparation

The human SNP data were downloaded from the HapMap Project (Phase II and Phase III) and the 1000 Genomes Project [22]. The projects collected genotypes instead of complete genomic sequences of individuals from different populations. We obtained the phased genetic variants in the MHC region (28,477,797bp - 33,448,354bp in chromosome 6 at 6p21.3) from the two projects (i.e. HapMap Phase III and 1000 Genomes) and the genome-wide SNP data of the European population (CEU) from HapMap Phase II release 22. The MHC region contains 166,053 SNPs, and overall 2,543,887 SNPs were collected from the whole human genome. The HapMap Phase II and III data were lift over to the human genome assembly GRCh37, Hg19 from the previous assembly of Build 36.1 (hg18) using the UCSC liftOver tool.

The LDsplit software [15], which is the implementation of the LDsplit algorithm [14], was used to calculate the association between sequence polymorphisms and meiotic recombination hotspots. The LDsplit algorithm divides a sample of haplotypes into two subgroups according to SNP alleles at a locus. The normalized difference $\Delta \rho =\frac {\rho _{0}-\rho _{1}}{\rho _{0}+\rho _{1}}$ was used to measure the difference in hotspot strength between the two subgroups of SNP alleles 0 and 1, where *ρ*_0_ and *ρ*_1_ present the recombination rates of the hotspot in the two different subgroups. For a specific SNP, the *p*-value that denotes the statistical significance of the hotspot-SNP association is estimated by testing against a simulated null distribution generated from the permutation test in order to avoid biased predictions [14].

**Fig. 1:**
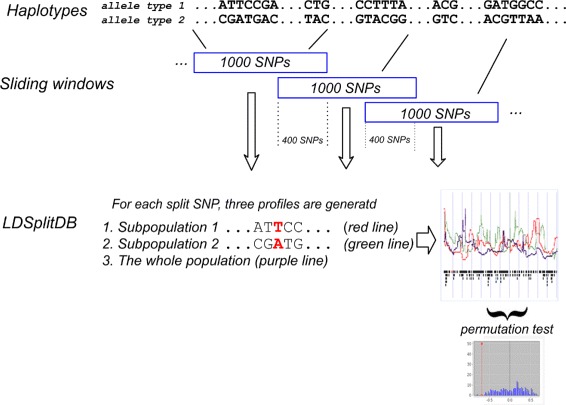
The workflow of data preparation for constructing LDSplitDB

Since LDsplit is based on LDhat which is computationally intractable to run on a whole chromosome, the haplotypes were cut into sliding windows of 1000 SNPs in length with an overlap of 400 SNPs between two consecutive windows (Fig. 1). Allele-specific recombination profiles were calculated for SNPs that have minor allele frequency (MAF) no less than 0.3. We call such SNPs as “split SNPs”. The permutation test is done by randomly splitting the haplotypes for 200 times in each window to simulate a null distribution for the calculation of *p*-values. To alleviate the heavy computational burden, we only calculated the recombination profiles of split SNPs in their sliding windows rather than the whole chromosome or MHC region, since the impact of SNPs on the recombination hotspots decays with increasing distances between the hotspots and SNPs [23–25].

Totally 8732 windows (1530 from HapMap Phase III across 10 populations and 7202 from the 1000 Genomes Project across 26 populations) in the MHC region and 4237 windows in CEU autosomes from HapMap Phase II were identified. The recombination profiles corresponding to different SNP alleles are stored in the LDSplitDB database. Three recombination profiles were provided for each split SNP, i.e. those of the whole sample and of the two subsamples divided by the alleles of the split SNP.

Several works on DNA methylation and histone modifications highlight epigenetic features of the meiotic recombination hotspots, suggesting crucial roles of epigenetics in recombination [26, 27]. ENCODE [19] and the NIH Roadmap Epigenomics Mapping Consortium [20] are two established sources of human epigenomic data, e.g. DNA methylation, histone modifications and chromatin accessibility. In addition, MethBase [28] is a central reference methylome database created from public BS-seq datasets, and it contains hundreds of methylomes from well-studied organisms. The databases mentioned above are integrated as public track hubs into our database to provide a wealth of auxiliary information for integrative analysis of SNPs and recombination hotspots.

### Database content and interface

LDSplitDB offers a user-friendly web interface to facilitate easy access to the database. There are mainly two query services: retrieving pre-computed recombination profiles and downloading a pre-computed lookup table with 400 haplotypes for running LDhat to estimate recombination rates. Moreover, a local UCSC Genome Browser [29] has been integrated in the web server to visualize the results. Other data resources, e.g. epigenomic data from ENCODE [19], are integrated into the auxiliary tracks.

To estimate recombination rates from large genotype data, we prepared a lookup table of 400 haplotypes for recombination estimation using LDhat which includes all possible two-locus haplotype configurations. Compared to the existing lookup tables of sizes no more than 192 [21], it satisfies the needs of analyzing larger human genetic variation data.

### Search features

**Fig. 2:**
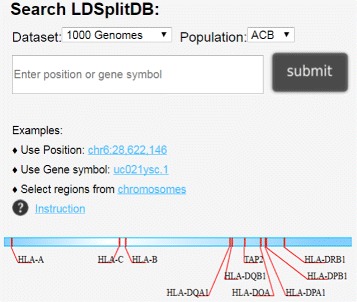
Illustration of a query service in LDSplitDB

The query service provides various modes of accessing to the three datasets, i.e. MHC region in the 1000 Genomes Project, MHC region in the HapMap Phase III dataset, and the genome-wide analysis of the HapMap Phase II data (European population). LDSplitDB supports queries using gene names (in UCSC IDs), or positions on a chromosome (Fig. 2). In addition, 10 most studied MHC genes, including HLA genes of classes I, II, and III, are plotted on a segment of chromosome 6. Users can click the genes to display the flanking recombination profiles. The results can be shown in the UCSC Genome Browser.

### Auxiliary tracks

To facilitate user’s investigation of the mechanisms for regulating meiotic recombination hotspots, related annotations have been integrated as auxiliary tracks. Epigenetic modifications, e.g. H3K4me3 [30, 31], H3K9ac [32] and H3K9me3 [26, 33], are well-known to be important for the regulation of recombination hotspots. Thus we collected datasets that have been demonstrated to be closely related to meiotic recombination from existing databases (details in “Data preparation” section), including ENCODE [19], the NIH Roadmap Epigenomics Mapping Consortium [20] and MethBase [28]. The selected public track hubs are displayed along with the main track of LDSplitDB using the same coordinates.

### Association studies

To calculate the *p*-value of the association between a hotspot and a split SNP, users need first locate the boundaries of the hotspot by selecting an area on the LDSplitDB channel in the UCSC Genome Browser. After selecting the hotspot region, the button labeled “Calculate *p*-value” can be clicked, and the calculated *p*-values will be shown in a table.

### Analysis tools

A list of useful tools is listed to facilitate the study of recombination events. The stand-alone package LDsplit [15] is a software tool to detect SNPs associated with meiotic recombination hotspots. It is the first computational method that explores large-scale genetic variation of recombination hotspots among individuals. CPLDhat [34] is an open-source Java program developed to estimate recombination rates more efficiently than the original LDhat algorithm. It contains two methods, i.e. CLDhat and PLDhat. Compared with LDhat, CLDhat enhances the time efficiency and user-friendliness by automatically predicting parameters and monitoring the mixing process. PLDhat employs parallel computation to further accelerate the CLDhat program. CLDhat and PLDhat provide faster methods for the estimation of recombination rates.

## Utility

The MHC region is well-known for its high gene density and polymorphism in the human genome. The polymorphisms in this region play a key role in susceptibility to immune diseases. It has been also suggested that different haplotypes could affect the frequencies and locations of recombination hotspots.

In this section, we describe two case studies to illustrate the potential applications of LDSplitDB to analyzing MHC region and revealing disease-associated recombination events.

### Hotspot-SNP association study

*DNA2* is one of the six meiotic recombination hotspots discovered in the class II region of MHC by sperm typing [24]. It is located in the non-coding intergenic region downstream of *HLA-DOA* (UCSC ID: uc010jui.3) which is the alpha subunit of the HLA class II complex. Jeffreys et al. [35] demonstrated a significant hotspot-SNP association between FG11 SNP (rs417812, position: 32,974,081bp) and the *DNA2* hotspot. FG11 is located within the *DNA2* hotspot. Here we use LDSplitDB to reproduce the experimental result by computational analysis.

**Fig. 3:**
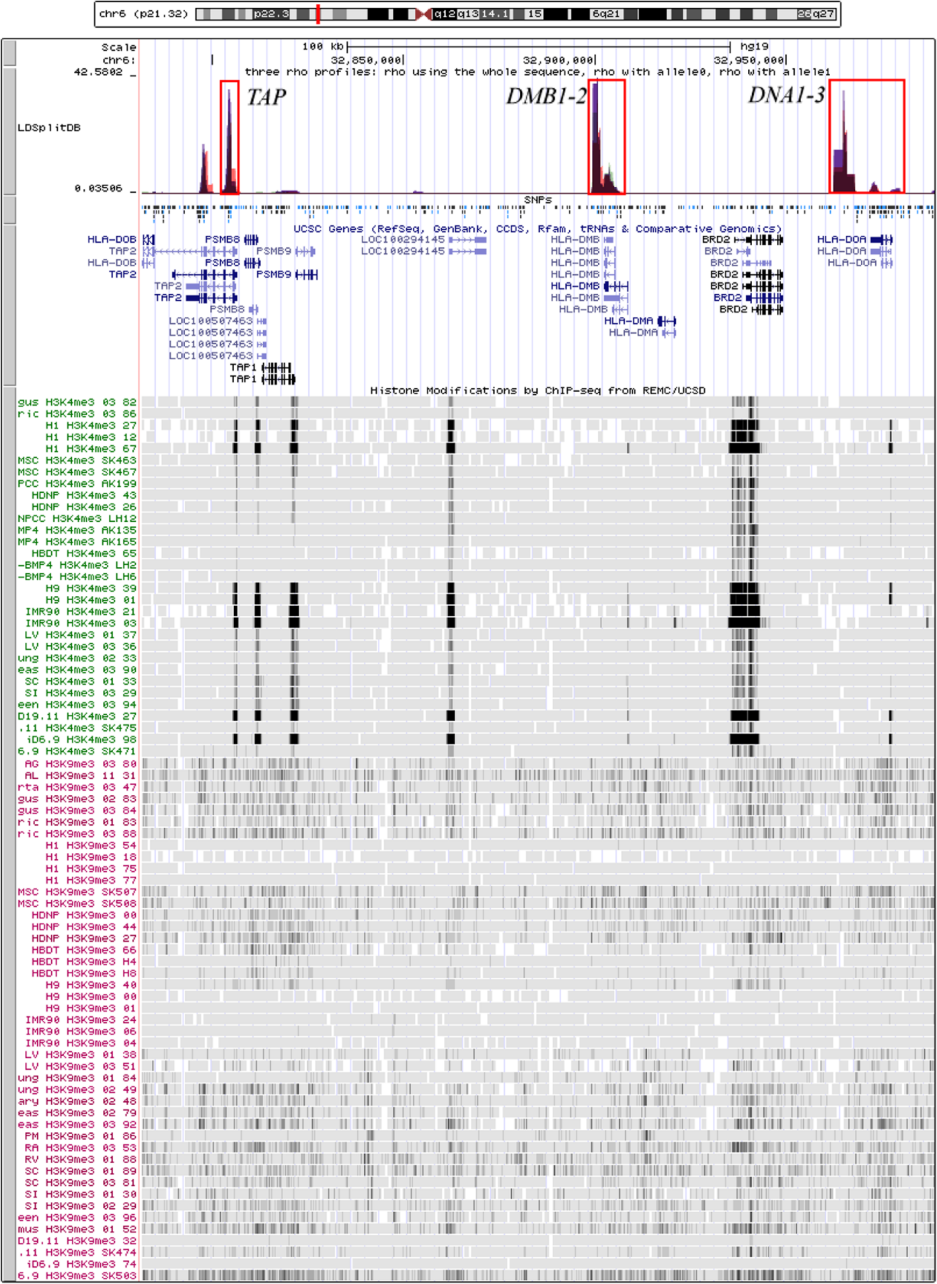
The locations of the six hotspots identified in a fragment of MHC by sperm typing [24] and histone modification profiles (H3K4me3, H3K9me3) from Roadmap Epigenomics Project

First, we located the gene *HLA-DOA* to search for the target *DNA2* hotspot using CEU data from the HapMap III data. Three DNA hotspots (*DNA1-3*), two DMB hotspots (*DMB1-2*) and the *TAP2* hotspot were identified (Fig. 3). We compared the H3K4me3 profiles from various cell lines with the H3K9me3 profiles. Most of the cell lines, especially embryonic stem cell lines (H1 and H9), show a high signal of H3K4me3 and a low signal of H3K9me3 indicating an active state of the chromatin which is a feature of active hotspots. We have also noticed the epigenetic differences between different types of cells [36].

*DNA2* is between *HLA-DOA* and *BRD2*. Then we selected the putative hotspot region. The *p*-value of the association between the FG11 SNP and the *DNA2* hotspot was 0.025859 suggesting a significant association.

### Study of recombination-related diseases

**Fig. 4:**
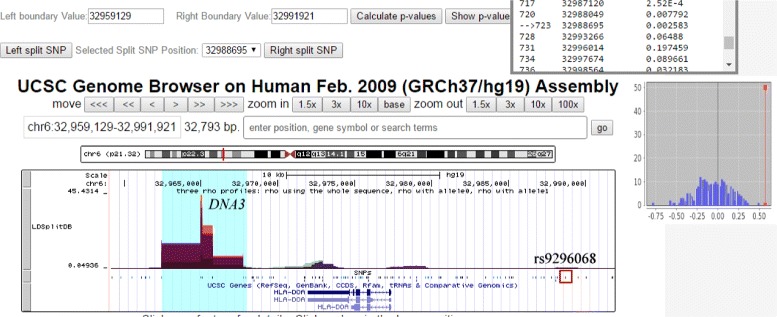
The association analysis of *DNA3* hotspot and related SNPs

Errors during meiotic recombination, e.g. extra DNA replication or deletion, could lead to serious birth defects. Childhood acute lymphoblastic leukemia (ALL) is a malignant disease occurring in children associated with certain human leukocyte antigen (HLA) alleles in MHC [37]. Thompson et al. [38] used LDsplit to study the association of 35 proximal SNPs with the *DNA3* hotspot including two previously validated SNPs. Their study indicated that meiotic recombination rates at *DNA3* could be influenced by sequence polymorphisms in the flanking regions which may contribute to disease susceptibility. Using LDSplitDB, we have replicated with ease some of their results, e.g. SNP rs9296068 is located at 32,988,695bp and has significant association with *DNA3* (p = 0.002) (Fig. 4).

## Discussion and conclusion

The availability of rapidly increasing data of genome-wide human genetic variation makes it possible to conduct large-scale association studies of human meiotic recombination hotspots. In this paper, we described LDSplitDB, a web-based database for the study of meiotic recombination hotspots. It contains large-scale allele-specific recombination profiles in the MHC region as well as a genome-wide map of hotspot-SNP association. In addition, the integration of auxiliary data from diverse sources would help users to study recombination hotspots from multiple perspectives and make novel discoveries. The integrative and interactive user interface provided by LDSplitDB can aid future experimental and computational studies to elucidate the regulatory mechanisms of meiotic recombination hotspots and their roles in human diseases.

The human SNPs data are increasingly expanded with more populations and haplotypes, and the new sequencing technology, e.g. single-cell whole-genome sequencing [39], can provide new data and insights. By integrating diverse types of information and supporting larger-scale computational inference of recombination rates, LDSplitDB paves the way for future knowledge discoveries from population genomics data of humans as well as other species.
